# Isolation and Identification of Efficient Malathion-Degrading Bacteria from Deep-Sea Hydrothermal Sediment

**DOI:** 10.3390/microorganisms10091797

**Published:** 2022-09-06

**Authors:** Ling Ma, Xin Dai, Guomin Ai, Xiaofang Zheng, Yanfeng Zhang, Chaozhi Pan, Meng Hu, Chengying Jiang, Li Wang, Zhiyang Dong

**Affiliations:** 1 State Key Laboratory of Microbial Resources, Institute of Microbiology, Chinese Academy of Sciences, Beijing 100101, China; 2 College of Life Sciences, University of Chinese Academy of Sciences, Beijing 100049, China; 3 Shenzhen Siyomicro BIO-Tech Co., Ltd., Shenzhen 518116, China; 4 State Key Laboratory of Developmental Biology of Freshwater Fish, Department of Microbiology, College of Life Science, Hunan Normal University, 36 Lushan Rd., Yuelu District, Changsha 410081, China

**Keywords:** bioremediation, deep-sea, malathion, *Pseudidiomarina* sp.

## Abstract

The genetic and metabolic diversity of deep-sea microorganisms play important roles in phosphorus and sulfur cycles in the ocean, distinguishing them from terrestrial counterparts. Malathion is a representative organophosphorus component in herbicides, pesticides, and insecticides and is analogues of neurotoxic agent. Malathion has been one of the best-selling generic organophosphate insecticides from 1980 to 2012. Most of the sprayed malathion has migrated by surface runoff to ocean sinks, and it is highly toxic to aquatic organisms. Hitherto, there is no report on bacterial cultures capable of degrading malathion isolated from deep-sea sediment. In this study, eight bacterial strains, isolated from sediments from deep-sea hydrothermal regions, were identified as malathion degradators. Two of the tested strains, *Pseudidiomarina homiensis* strain FG2 and *Pseudidiomarina* sp. strain CB1, can completely degrade an initial concentration of 500 mg/L malathion within 36 h. Since the two strains have abundant carboxylesterases (CEs) genes, malathion monocarboxylic acid (MMC α and MMC β) and dibasic carboxylic acid were detected as key intermediate metabolites of malathion degradation, and the pathway of malathion degradation between the two strains was identified as a passage from malathion monocarboxylic acid to malathion dicarboxylic acid.

## 1. Introduction

Malathion (S-(1,2-dicarbethoxyethyl)-*O*, *O*-dimethyldithiophosphate) is an organophosphorous pesticide with highly selective toxicity that has not only been widely used to protect field and horticulture crops in agriculture, but also has been sprayed to control pests and sustain hygiene in public recreation and residential areas [1]. Although it is effective in pest control, malathion is toxic to non-targeted living organisms including human beings, beneficial insects, snails, micro crustaceans, fish, birds, amphibians, soil microorganisms, and so on [1,2].

As a nerve poison, malathion irreversibly inhibits the enzyme acetylcholinesterase (AChE) [3,4], builds up free acetylcholine in nervous tissues, and ultimately causes serious neurological health disease. It impairs the central nervous system of invertebrates and immune system of higher vertebrates, reproductive functions of vertebrates, and the adrenal glands and tissues of fish [3,5,6,7]. Even at a low level of 0.06 ppb, malathion is highly toxic to some freshwater fish. Further, malathion is affirmed to be genotoxic, and exposure to fish tissues has shown damage to DNA [8]. Malathion is also a model for the enzymatic hydrolysis of the neurotoxic agent VX, the organophosphorus hydrolase (OPH) enzyme, 72 kDa and a dimeric entity consisting of two identical subunits of 336 amino acid residues, derived from *Pseudomonas diminuta* that hydrolyzes the P–S bond of malathion. The hydrolysis results in the complete loss of AChE inhibitory potency [9]. High levels of malathion in soil or water bodies contaminate the natural environment and cause ecological imbalance, resulting in the loss of marine diversity [10].

Microorganisms have gained much attention due to their potential applications in bioremediation of environment pollutants [11]. Microbial bioremediation of malathion has been reported to be highly promising for both *in-situ* and *ex-situ* treatment owing to its cost effectiveness and environment-friendly nature. Reported malathion-degrading agents include bacterium strains (*Rhizobium*, *Pseudomonas* sp., *Pseudomonas putida*, *Micrococcuslylae*, *Pseudomonas aureofaciens*, *Acetobacter liquefaciens*, *Bacillus cereus*, *Acinetobacter baumannii*, *Brevibacillus, and Lysinibacillus,* among others) [10,12,13,14,15,16,17,18,19] and fungal strains (*Aspergillus Oryzae, Asperigillus niger, Penicillium notatum,* and *Rhizoctonia solani*, among others) [20]. Among them, the *Pseudomonas* sp. strain IS2 completely degraded 50 mg/L malathion within 6 days [20], while the *Bacillus licheniformis* strain ML-1 was able to degrade 78% of fed 350 µg/L malathion within 5 days [21]. The malathion degradation rate of *Acinetobacter baumannii* strain 5E and the *Acidothiobacillus ferroxidans* strain 6F was recorded between 30–40% within 36 h [10]. The *Brevibacillus* sp. strain KB2 degraded 36.22% of the malathion at an initial concentration of 72.5 mg/L in 7 days [16]. For malathion metabolism, the fungi are highly efficient at malathion degradation; for example, *Fusarium oxysporium* f. sp. can degrade almost 60% malathion at an initial concentration of 500 mg/L in 0.5 h [22]. The degradation potential of malathion depends mainly on the microorganism enzymatic activity. Enzymes are biocatalysts that can accelerate certain biological reactions by reducing the activation energy of the reaction rate in organisms [23]. Enzymes involved in the degradation of malathion include hydrolases (organophosphate hydrolases and organophosphorus acid anhydrase) [24], esterases (carboxylesterase) [19], phosphatases [18,25], and oxidoreductases [14,26]. Malathion is degraded by carboxylesterases to its monoacid and diacid derivatives; this is the main metabolic mechanism for the degradation of malathion by microorganisms. In past studies, carboxylesterase was isolated from *Bacillus cereus*, *Brevibacillus*, and *Lysinibacillus*, and a novel carboxylesterase enzyme was isolated from *Acinetobacter baumannii* as well [16,17,19,27].

Isolation and characterization of metabolic malathion-degrading bacteria is not only of utmost importance to develop an efficient biological treatment system using pure culture for malathion-contaminated environments, but is also a sustainable, cost-effective, and practical technique for the degradation of pesticides in contaminated environments. Abundant and diverse marine microbial genetic and metabolic resources play an important role in maintaining the ecological balance of the ocean. Marine microorganisms participate in Earth’s element cycles through the production and consumption of organic matter. Particularly, sulfur and phosphorus are essential constituents in biomass and crucial players in climate processes. The sulfur and phosphorus cycle is affected by microbial activities [28], because marine microorganisms contain huge phosphorus–sulfur metabolism potential. However, due to the insufficient sampling and lack of understanding on culture conditions of marine microorganisms, the phosphorus and sulfur research on marine microorganisms is not very extensive.

As of 2022, malathion degradation by deep-sea bacteria culture has yet to be reported. In this study, eight strains isolated from deep-sea paleo-hydrothermal sediments were identified as malathion degradators: *Idiomarina andamanensis* strain 58-SNN-6, *Idiomarina loihiensis* strain 07WCL4, *Idiomarina loihiensis* strain 07WCL3, *Pseudidiomarina gelatinasegens* strain 58-SNN-9, *Idiomarina loihiensis* strain R30 and *Idiomarina abyssalis* strain CG1, *Pseudidiomarina homiensis* strain FG2, and *Pseudidiomarina* sp. strain CB1. Particularly, two highly efficient malathion-degrading strains, *P. homiensis* strain FG2 and *Pseudidiomarina* sp. strain CB1, were able to completely degrade fed malathion within a short time. The key intermediate metabolites were characterized.

## 2. Materials and Methods

### 2.1. Malathion

Stock solution of 50 g/L malathion (Dr. Ehrenstorfer, Augsburg, Germany) dissolved in dimethyl sulfoxide (DMSO) was supplemented to experimental medium at a ratio of 1% (*v/v*) so that the applied concentration in medium was 500 mg/L.

### 2.2. Deep-Sea Hydrothermal Region Samples and Experimental Media

Three sediment samples were obtained from a deep-sea hydrothermal region. Sample R8 (13°13′63″ N, 114°49′58″ E, 3014 m water depth) and R9 (15°3′4.363″ N, 116°52′29″ E, 988 m water depth) were from South China Sea paleo-hydrothermal region, while sample DY3907 (34°58′1.452″ S, 54°12′51.4″ E, 3270 m water depth) was from Southwest Indian Ridge hydrothermal region. The sediment samples were stored in sterile tubes at 4 °C before use.

Marine 2216E medium composed of 5 g/L tryptone, 1 g/L yeast extract, 0.1 g/L Iron (III) citrate, 23.477 g/L NaCl, 3.917 g/L Na_2_SO_4_, 4.981 g/L MgCl_2_, 1.102 g/L CaCl_2_, 0.192 g/L NaHCO_3_, 0.664 g/L KCl, 0.006 g/L KBr, 0.02 g/L H_3_BO_3_, 0.024 g/L SrCl_2_, 0.003 g/L NaF was used for strains enrichment cultivation and screening. M1 medium containing 18 g/L sea salt (Sigma, Saint Louis, MO, USA), 0.4 g/L yeast extract, 0.3 g/L glucose, and 1 g/L (NH_4_)_2_SO_4_ was used for malathion-degrading bacterial isolation and corresponding metabolites detection.

### 2.3. Bacterial Isolation from Deep-Sea Hydrothermal Region Samples

First, the three sediment samples from deep-sea hydrothermal region were incubated in 2216E liquid medium at 28 °C in oscillation incubator (ZHICHU, Shanghai, China) at 200 rpm for enrichment culture. After 2 days, serial dilution and plating method on 2216E agar medium were used for isolation of the strains. Single colonies were picked and streaked on solid medium plates followed by incubation at 37 °C for 48 h. Separate colonies were sub-cultured on marine 2216E agar plates till pure cultures were obtained. About 40 purified colonies isolated from three deep-sea sediment samples were preserved in 20.0% (*v/v*) glycerol–water solution at −80 °C for further study.

### 2.4. Isolation and Screening of Biodegrading Strains

The isolated bacteria were cultured in 10 mL 2216E medium (100 mL shaking flask) at 28 °C and 200 rpm until OD_600_ reached 0.8, and 5 mL culture medium was collected and centrifuged at 4 °C and 4500 rpm for 10 min by a centrifuge (Thermo, Waltham, MA, USA). After supernatant was discarded, cell pellet was resuspended cell in 1 mL M1 medium. Centrifugation and cell washing were repeated 1–2 times.

For the isolation of malathion degrading bacteria, the 300 µL bacteria solution (4 OD) were first inoculated into 10 mL M1 medium containing 500 mg/L malathion, then cultivated in dark at 28 °C and 200 rpm for 48 h. Sterile controls were conducted and all the experiments were triplicated.

After 48 h cultivation, 10 mL n-hexane was added to extract residual malathion and its metabolites in medium. Following homogenization by a vortex mixer (Scientific Industries, Bohemia, NY, USA) the mixture stood for 2 h at 4 °C, then 1 mL of upper layer solution (n-hexane) were transferred into an EP tube and concentrated by rotary evaporation in a freeze concentrator at 30 °C for 10 min; 1 mL acetonitrile was added to the EP tube and mixed well for further detection of the concentration of residual malathion by high-performance liquid chromatography.

Concentration of malathion was determined by HPLC (high-performance liquid chromatograph, Agilent, Santa Clara, CA, USA) system equipped with a C18 reversed-phase analytical column (ZORBAX, 4.6 mm × 100 mm dp = 3.5 µm) plus suitable guard column and a diode-array detector. Flow rate of mobile phase (acetonitrile: water/50:50) was fixed at 1.0 mL/min, 10 µL acetonitrile extract was injected, and detection wavelength was set as 215 nm. The cultures with higher degradation rate (calculated as formulation 1) of malathion were stored on nutrient-agar slants covered with glycerol at 4 °C.

(1) $$\mathrm{Degradation}\;\mathrm{Rate}\;\%=\frac{\mathrm{Sterile}\;\mathrm{control}\;\mathrm{content}\;\mathrm{of}\;\mathrm{malathion}\text{-}\mathrm{experiment}\;\mathrm{content}\;\mathrm{of}\;\mathrm{malathion}}{\mathrm{Sterile}\;\mathrm{control}\;\mathrm{content}\;\mathrm{of}\;\mathrm{malathion}}\times 100$$

### 2.5. Identification and Characterization of Biodegrading Strains

The identification and characterization of biodegrading strains were carried out by morphological and phylogenetic analyses. The light microscope (LEICA DM500) and a scanning electron microscope were used to examine the colony features and the cell morphological structure, respectively. Gram-staining was conducted by the method described by Hucker et al. [29] and observed by light microscope.

### 2.6. Growth Study

Cell growth of pesticide-degrading microorganisms in M1 medium containing 500 mg/L malathion was evaluated by growth curved.

In a sterile conical flask holding 10 mL of M1 medium containing malathion (500 mg/L), an aliquot (300 µL, 4 OD) of bacterial suspension was inoculated and incubated in an oscillation incubator (ZHICHU ZQZY-CF8, Shanghai, China) in dark at 200 rpm and 28 °C. The OD_600_ was measured at different time intervals (2 h, 6 h, 10 h, 12 h, 18 h, 24 h, and 36 h) by an INESA UV/VIS spectrophotometer L5S (INESA, Shanghai, China). The pH of growth media was adjusted to pH 7. The growth curve was plotted by determining the optimal culture time of each biodegrading strain at 28 °C. All the experiments were triplicated, and a negative control was set as identical medium without inoculation.

### 2.7. DNA Extraction, PCR Amplification, and Sequencing of 16S rRNA

Genomic DNA was extracted and purified with a bacterial genomic DNA extraction kit (Tiangen, Beijing, China). The 16S rDNA gene was amplified by PCR using bacterial universal primers: 27F (5′-AGAGTTTGATCCTGGCTCAG-3′) and 1492R (5′-ACGGCTACCTTGTTACGACT-3′). PCR reaction mixture contained 1 µL of 50 ng genomic DNA, 2 µL of primer pair, and 12.5 µL of 2 × Lamp Master Mix (Vazyme, Nanjing, China), with deionized H_2_O adjusting the final volume to 25 µL. PCR amplification was performed for 35 cycles after an initial denaturation step for 5 min at 95 °C. Each cycle consisted of denaturation at 94 °C for 30 s, annealing at 50 °C for 30 s, and extension at 72 °C for 30 s. The obtained sequences were blasted with other known sequences in the National Center for Biotechnology Information (NCBI) database and aligned with the related strains using ClustalW in Mega version X.

### 2.8. DNA Extraction, Genome Sequencing, and Assembly

For genome sequencing, genomic DNA of strains with malathion degradation were extracted by a genomic DNA extraction kit (Tiangen, Beijing, China). The genomes of the strains were sequenced by the Illumina Hiseq system with 500/350 bp paired-end libraries. De novo assembly was performed using SPAdes [30], and draft genomes were constructed. Whole-genome average nucleotide identity (ANI) was estimated by the JSpeciesWS online service (http://jspecies.ribohost.com/jspeciesws/ (accessed on 15 November 2021)), and digital DNA–DNA hybridization (dDDH) and G + C difference values for whole genome sequences between the two strains, and *P. homiensis* PO-M2^T^ were conducted through DSMZ’s online service (http://ggdc.dsmz.de (accessed on 15 November 2021)).

### 2.9. Phylogenetic Analysis

Published sequences of 40 bacterial strains (*Idiomarina* family) and their accession numbers were obtained from GenBank, and a phylogenetic tree was constructed to compare their sequences of 16S rRNA gene with eight representative malathion-degrading bacterial strains (FG2, CB1, CG1, 58-SNN-6, 58-SNN-9, R30, 07WCL3, and 07WCL4). Sequencing alignment was performed using the CLUSTALW program, and following the gene ration of 1000 bootstrap replicates, MEGA 7.0 was used to construct a phylogenetic tree using the neighbor-joining technique. Phylogenetic tree was displayed using Tree view.

### 2.10. Identification of Metabolites Produced from Malathion Biodegradation

Strain FG2 and strain CB1 were incubated in the M1 medium containing malathion (500 mg/L) for 24 h. Cells were removed by centrifugation, and an aliquot (1 mL) was then taken from the supernatant and mixed with 1 mL acetonitrile; then, 10 μL sample was injected for LC-MS/MS analysis.

LC-MS/MS analyses were conducted in an Agilent 1260 Infinity II LC System (Santa Clara, CA, USA) comprising an ultra-HPLC instrument coupled to an Agilent 6460 triple quadrupole mass spectrometer. Mobile phase was composed of 0.1% formic acid and 2% methanol in water (solvent A) and pure acetonitrile (solvent B). The binary pump delivered the mobile phase at the gradient of initial 95% A-5% B, holding at 95% A-5% B for 2 min, programming to 10% A-90% B over 29 min, and holding at 10% A-90% B for 27 min. The single run lasted 44 min. Prior to next injection, 15 min equilibrium time was set. The temperature of the C18 hyper purity column (100 mm × 2.1 mm i.d., particle size 3.5 µm) was kept at 35 °C. The mass spectrometer used a jet stream (electrospray) ionization source that was operated at a gas temperature of 350 °C, gas flow 12 L/min, and nebulizer 35 psi. The other conditions were capillary voltage 4000 V and nozzle voltage 500 V. Data acquisition was performed using the Mass Hunter software (Santa Clara, CA, USA). Optimization of the transitions three molecules mixture were injected individually into the LC-MS/MS system.

## 3. Results

### 3.1. Isolation and Degradation Activity of Malathion-Degrading Bacteria

**Figure 1:**
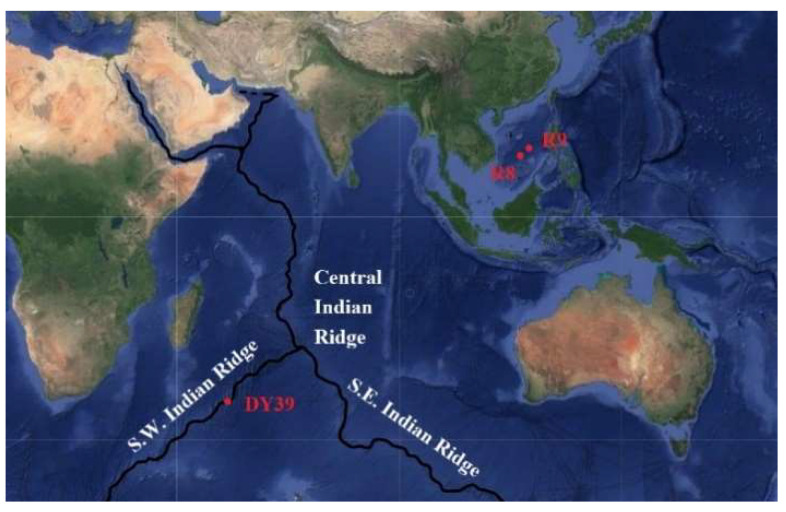
The geographic map of sediment sample collection sites.

**Figure 2:**
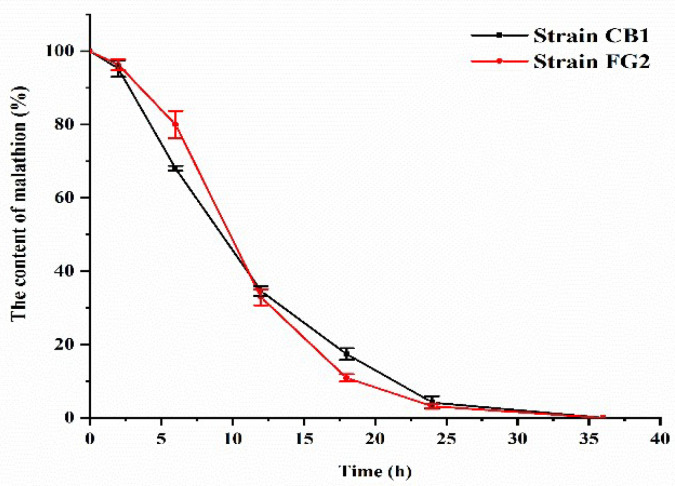
Biodegradation of malathion by strain FG2 and strain CB1 in M1 medium containing 500 mg/L malathion. Data are presented as mean and standard error (*n* = 3).

**Figure 3:**
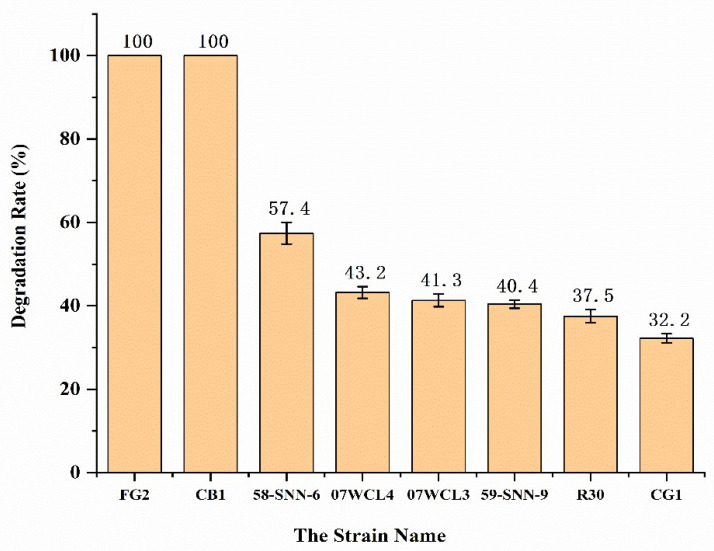
Biodegradation rates of malathion by eight strains isolated from sediment samples from the deep-sea source. Degradation experiments were conducted in 100 mL Erlenmeyer flasks containing 10 mL M1 medium with a mixture of 500 mg/L malathion as carbon sources. The residual malathion was quantified by HPLC; the degradation percentage was the average of triplicate repeats at 48 h.

Forty colonies were isolated from three sediment samples from deep-sea hydrothermal region (Figure 1). Eight bacterial isolates were able to grow in M1 containing 500 mg/L malathion. According to HPLC analysis, all these eight strains were successfully screened as malathion-degrading. Further, all these eight bacterial isolates were able to grow in M1 containing 500 mg/L malathion. After 36 h incubation, strain FG2 and strain CB1 showed complete degradation of malathion (Figure 2). Strains 58-SNN-6, 07WCL4, 07WCL3, 58-SNN-9, R30, and CG1 were able to degrade malathion with degradation rates of 57.4%, 43.2%, 41.3%, 40.4%, 37.5%, and 32.2%, respectively, after 48 h of incubation (Figure 3).

### 3.2. Taxonomy of Malathion-Degrading Bacteria

**Figure 4:**
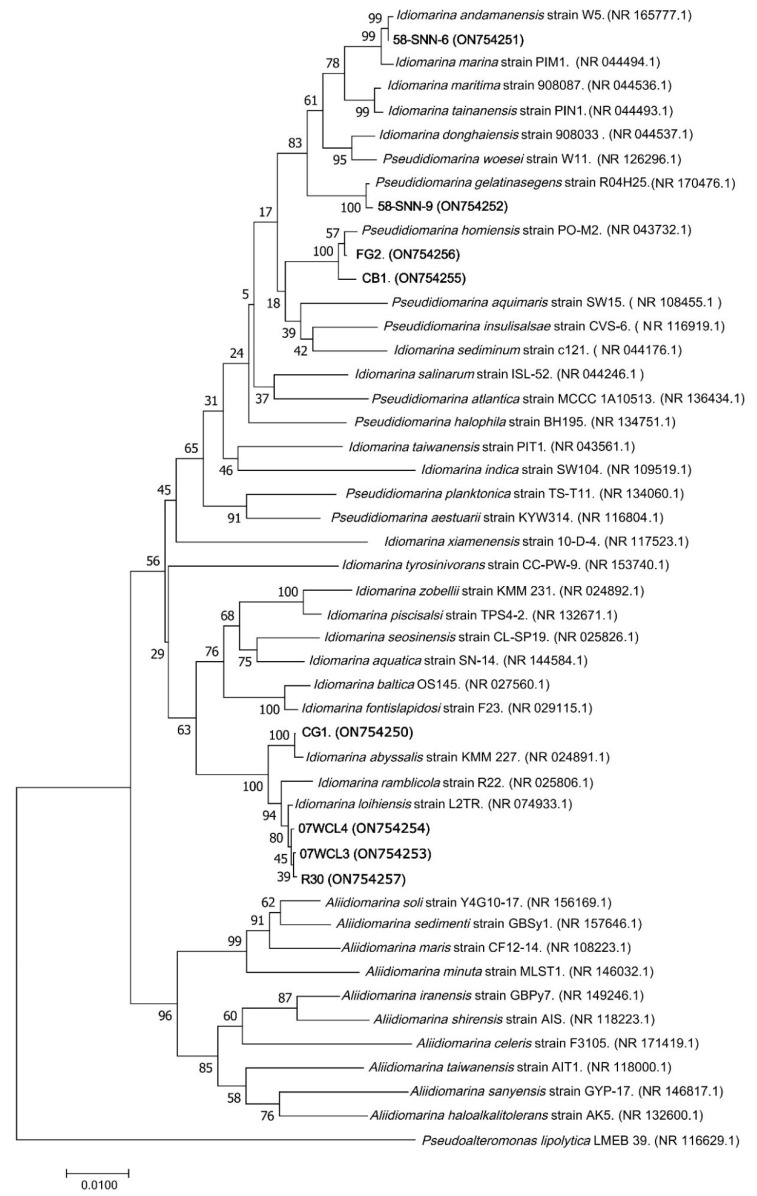
Unrooted phylogenetic tree reconstructed by the neighbor-joining method based on 16S rRNA gene sequences of strains CB1, FG2, CG1, 58-SNN-6, 58-SNN-9, 07WCL3, 07WCL4, R30, and other reference species in the family *Idiomarina*, demonstrating the phylogenetic relationship of the isolates. Bootstraps were calculated for 1000 replications and shown at branch nodes. Bar, 0.01 substitutions per nucleotide position. GenBank accession numbers are given in parentheses.

Comparative analysis of 16S rRNA gene sequences from NCBI showed that strain FG2 and CB1 were closest to *P. homiensis* PO-M2^T^ with sequence similarity above 99.59%, while strain 58-SNN-6 was closest to *Idiomarina andamanensis* W5^T^ (99.78% sequence similarity), strain 07WCL4 was closest to *Idiomarina loihiensis* L2-TR^T^ (99.86% sequence similarity), strain 07WCL3 was closest to *Idiomarina loihiensis* L2-TR^T^ (99.93% sequence similarity), strain 58-SNN-9 was closest to *Pseudidiomarina gelatinasegens* R04H25^T^ (99.86% sequence similarity), strain R30 was closest to *Idiomarina loihiensis* L2-TR^T^ (99.93% sequence similarity), and strain CG1 was closest to *Idiomarina abyssalis* KMM 227^T^ (99.86% sequence similarity). The NJ phylogenetic tree constructed by 16S rRNA gene sequences of the isolates and their relatives showed that these strains formed a distinct clade (Figure 4).

### 3.3. Genome Analysis

The assembled draft genome of FG2 and CB1 contained six contigs each, the sizes of which were 2.57 and 2.64 Mbp, and the G + C contents of 50.04 and 49.81 mol%, respectively. The genomes of FG2 and CB1 contained 2389 and 2457 predicted genes, respectively, 49 and 52 tRNA genes, and three rRNA genes each, were identified, respectively.

Compared by the genomes, the ANI value between CB1 and *P. homiensis* PO-M2^T^ was 94.53%, below the 95–96% interspecies threshold, and the ANI value between FG2 and *P. homiensis* PO-M2^T^ was 97.66%. The dDDH value between CB1 and *P. homiensis* PO-M2^T^ was 56.1%, below the threshold of 70% proposed by Moore et al. for bacterial species classification [31], and the dDDH value between FG2 and *P. homiensis* PO-M2^T^ was 79.3%. The results of the genome analysis were consistent with the position of the CB1 and FG2 in the tree and further confirmed that strain CB1 represents an unreported species belonging to the genus *Pseudidiomarina* and that strain FG2 signals a published species belonging to *P. homiensis.* Therefore, strain CB1 and strain FG2 represented two distinct genospecies of the genus *Pseudidiomarina.* Genome annotation showed that two strains have abundant carboxylesterases (CEs) genes.

### 3.4. Estimation of Bacterial Growth Rate and Change of pH during Malathion Degradation

**Figure 5:**
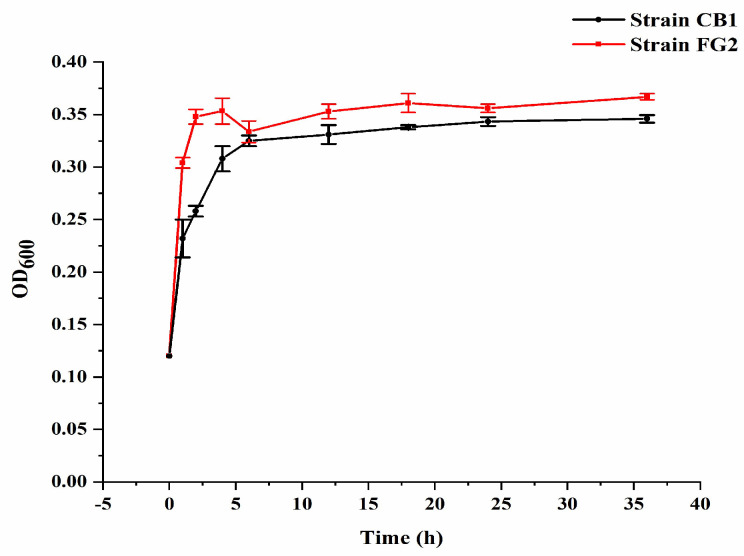
The growth of strain FG2 and strain CB1 and pH change in M1 medium. Bacterial growth was monitored for 36 h at different time intervals (2 h, 4 h, 6 h, 12 h, 18 h, 24 h, and 36 h). Data is presented as mean and standard error of three independent observations.

**Figure 6:**
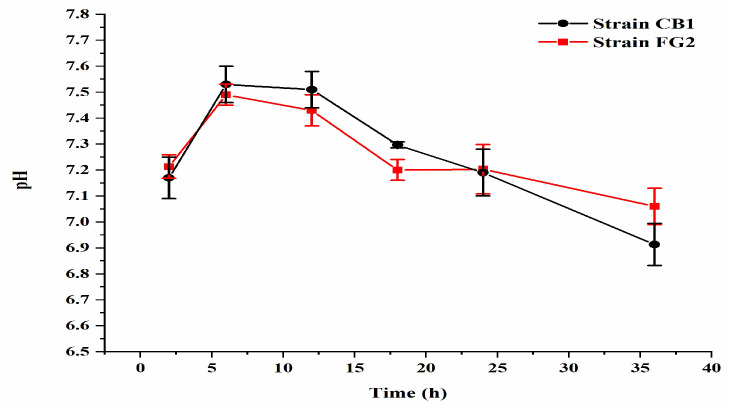
The pH of strain FG2 and strain CB1 in M1 medium. Bacterial growth was monitored for 36 h at different time intervals (2 h, 6 h, 12 h, 18 h, 24 h, and 36 h). Data is presented as mean and standard error of triplicates.

In M1 medium with 500 mg/L malathion, *P. homiensis* strain FG2 and *Pseudidiomarina* sp. strain CB1 attained the highest OD_600_ of 0.35 and 0.32, respectively (Figure 5), at 28 °C after 8 h, accompanied by the pH fluctuation from 7.0 to 7.5 during the first 10 h and the pH drop from 7.5 to 7.0 after 10 h (Figure 6).

### 3.5. Identification and Characterization of Efficient Malathion-Degrading Strains

**Figure 7:**
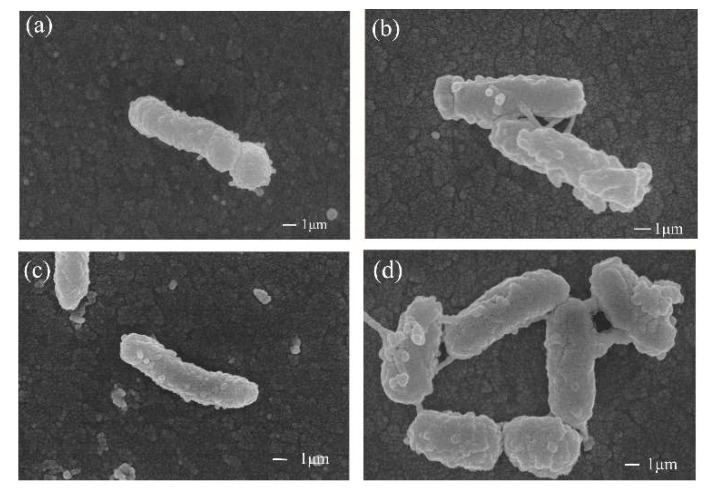
Scanning electron micrographs of strain FG2 and strain CB1 in different medium for 10 h. (**a**) Strain CB1 in 2216E medium; (**b**) strain CB1 in M1 medium containing 500 mg/L malathion. Cells of CB1 are regular rod morphotype with protuberances on the surface. (**c**) Strain FG2 in 2216E medium; (**d**) strain FG2 in M1 medium containing 500 mg/L malathion; cells of FG2 are regular rod morphotype with protuberances on the surface.

Two efficient malathion-degrading strains FG2 and CB1 were both Gram-negative bacteria. Cells of strain FG2 and strain CB1 were both 10.0 × 1.0 µm and were both regular rod morphotype with rough surface in 2216E medium for 10 h, while in M1 medium containing 500 mg/L malathion for 10 h, the surface of cells of strain FG2 displayed more long protuberances and sticked to each other (Figure 7).

### 3.6. Identification of Metabolites Produced from Malathion Biodegradation

Since genome annotation showed that *P. homiensis* strain FG2 and *Pseudidiomarina* sp. strain CB1 have abundant carbohydrate esterases genes, it is possible that the metabolites of malathion degradation catalyzed by carbohydrate esterase are monoacid and diacid derivatives.

By LC-MS/MS analysis, three peaks of malathion metabolites standard were identified, and they matched well with the mass spectrum pattern of malathion monocarboxylic acid (MMC α and MMC β) and dibasic carboxylic acid standard. Malathion monocarboxylic acid showed a parent ion peak at *m*/*z* 303.1 (retention time: 17.63 min (MMC α), 17.83 min (MMC β)) and a parent ion peak at *m*/*z* 275 for malathion dicarboxylic acid (MDC).

By LC-MS/MS analysis and by applying high resolution mass spectrometry in positive ionization mode with MS fragmentation, three malathion degradation product peaks were identified from both *P. homiensis* strain FG2 and *Pseudidiomarina* sp. strain CB1. The mass spectrum pattern showed a molecular ion peak at *m*/*z* [M–H]^+^ 303.1 with retention time of 17.63 min and 17.83 min, which are consistent with the molecular formula of malathion monocarboxylic acid homolog (MMC α and MMC β), C_8_H_15_O_6_PS_2_; the other molecular ion peaks were at *m*/*z* [M–H]^+^ 275 with retention time of 13.2 min, which is consistent with the molecular formula of malathion dicarboxylic acid (MDC), C_6_H_11_O_6_PS.

## 4. Discussion

Sulfur and phosphorus are essential components in all creatures and indispensable nutrient elements for living organisms. Sulfur-redox and phosphorus-redox microorganisms are ubiquitous in various environmental niches and have diverse metabolic pathways, while the balance between sulfur and phosphorus compounds depends on various sulfur-transforming and phosphorus-transforming reactions and metabolic pathways in the microbial metabolic network. There has long been an abundant phosphorus and sulfur circulation system in the ocean: the ocean sediments are, by far, the largest known stock in the biogeochemical cycles of phosphorus [32]. While there are distinctive features between the marine environment and the terrestrial environment, perhaps there are phosphorus and sulfur circulation methods and microorganisms that metabolize phosphorus and sulfur in the marine environment that have not yet been found on land.

This study isolated and identified two metabolic malathion bacteria (*P. homiensis* FG2 and *Pseudidiomarina* sp. CB1) from deep-sea sediments of a paleo-hydrothermal region. In this study, cultural medium supplemented with 0.04% yeast extract and 0.03% glucose [16] greatly promoted the metabolic efficiency of malathion. Two bacterial strains could degrade 100% of fed 500 mg/L malathion within 36 h. This is the first study that successfully isolated and identified *Pseudidiomarina* sp. as capable of degrading malathion. 

Regards as initially fed malathion concentration, 500 mg/L in this study is as several folds as applied in other studies, highlighting two malathion-degradation strains (FG2 and CB1) could adapt to the high concentration of malathion. Under similar culture conditions (MSM with the same 0.04% yeast extract and 0.03% glucose), *Brevibacillus* sp. strain KB2 and *Bacillus cereus* strain PU merely degraded by 36.22% and 49.31% within 7 days [16], which is inferior to CB1 and FG2 strains. 

This study also reported that a variety of other *Pseudidiomarina* sp. and *Idiomarina* sp. strains have the ability to degrade malathion. This is not yet covered by other studies, and these results provide ideas regarding the metabolism of organophosphorus compounds represented by malathion by *Pseudidiomarina* sp. and *Idiomarina* sp. bacteria. In two different media, 2216E medium and M1 medium, containing 500 mg/L malathion, there are differences in the surface morphology of strain FG2 and strain CB1. In the M1 medium containing 500 mg/L malathion, the surfaces of the two strains displayed more long protuberances and sticks; these long protuberances might be enzymes promoting the metabolism of malathion, which may be a topic for future research.

In this study, the draft genomes of malathion-degrading strain FG2 and CB1 showed a large number of sequences encoding carboxylesterases (CEs). Carboxylesterases (EC 3.1.1.1) are one of the major lipolytic enzymes responsible for hydrolysis. They can efficiently hydrolyze a large number of structurally diverse compounds with a specific functional group such as carboxylic acid esters, amides, as well as some thioesters [33]. Malathion was degraded to its monoacid and diacid derivatives through carboxylesterase, and this is in line with the reported mechanism of malathion degradation in microbes [16,17,27]. Based on the metabolites of malathion monocarboxylic acid (MMC α and MMC β) and malathion dicarboxylic acid that were identified by LC-MS/MS, the results indicate that the metabolic pathway of malathion degradation for *P. homiensis* strain FG2 and *Pseudidiomarina* sp. strain CB1 occurs through malathion monocarboxylic acid to malathion dicarboxylic acid. As the reaction progressed, the content of MMC α accumulated slightly, while the content of MMC β decreased and MDC increased, indicating that MMC α could be a self-hydrolyzed product of malathion and that MMC β could be hydrolyzed to produce MDC.

Based on the fact that (i) the predominant biodegradation pathway for malathion involves the formation of monocarboxylic acid (MMC α and MMC β) and (ii) malathion dicarboxylic acid metabolites via carboxylesterase activity, as well as genome annotation of *P. homiensis* strain FG2 and *Pseudidiomarina* sp. strain CB1, contained enriched carboxylesterases (CEs) genes, we conclude that carboxylesterases (CEs) genes are involved in the metabolism of malathion compounds and may play a key role in the metabolism of malathion. Nonetheless, the metabolic effect of different types of carboxylesterases (CEs) on malathion is also different, which deserves further exploration.

## 5. Conclusions

In this study, eight strains from sediment samples collected from South China Sea paleo-hydrothermal region and Southwest Indian Ridge hydrothermal region were able to degrade malathion. Among them, *Idiomarina andamanensis* strain 58-SNN-6, *Idiomarina loihiensis* strain 07WCL4, *Idiomarina loihiensis* strain 07WCL3, *Pseudidiomarina gelatinasegens* strain 58-SNN-9, *Idiomarina loihiensis* strain R30, and *Idiomarina abyssalis* strain CG1 were able to degrade malathion with degrade rate of 57.4%, 43.2%,41.3%, 40.4%, 37.5%, and 32.2%, respectively, after 48 h of incubation in in M1 medium containing 500 mg/L malathion.

The two strains with the most significant degradation efficiency were *P. homiensis* strain FG2 and *Pseudidiomarina* sp. strain CB1. Two efficient malathion-degradation bacterial strains can completely degrade 500 mg/L malathion within 36 h. *P. homiensis* strain FG2 and *Pseudidiomarina* sp. strain CB1 are Gram-negative bacteria that are rod-shaped with cross-linked cilia on the surface.

According to LC-MS/MS analysis, key intermediate metabolites of malathion were identified as monocarboxylic acid (MMC α and MMC β) and malathion dicarboxylic acid in *P. homiensis* strain FG2 (Figure S4) and *Pseudidiomarina* sp. strain CB1 (Figure S5).

## Data Availability

*Pseudidiomarina homiensis* strain FG2 genome sequencing and assembly: JANQBU000000000-BioProject-NCBI (nih.gov). *Pseudidiomarina* sp. CB1 genome sequencing and assembly: JANTPU000000000-BioProject-NCBI (nih.gov).

## Supplementary Materials

The following supporting information can be downloaded at: https://www.mdpi.com/article/10.3390/microorganisms10091797/s1, Figure S1: Gram staining of strain FG2 and strain CB1. (a) Gram-staining of strain FG2, Gram-negative rods. (b) Gram-staining of strain CB1, Gram-negative rods. Figure S2: Scanning electron micrographs of strain CB1 in different medium for 10 h. (a,b) Strain CB1 in 2216 E medium; (c,d) Strain CB1 in M1 medium containing 500 mg/L malathion. Figure S3: Scanning electron micrographs of strain FG2 in different medium for 10 h. (a,b) Strain FG2 in 2216 E medium; (c) Strain FG2 in M1 medium containing 500 mg/L malathion. Figure S4: Mass spectrum chart of metabolites of malathion by strain FG2, (a) malathion monocarboxylic acid (α type); (b) malathion monocarboxylic acid (β type); (c) malathion dicarboxylic acid. Figure S5: Mass spectrum chart of metabolites of malathion by strain CB1; (a) malathion monocarboxylic acid (α type); (b) malathion monocarboxylic acid (β type); (c) malathion dicarboxylic acid. Information: Bioproject ID of strain FG2 and strain CB1.

## References

[1] Singh B., Kaur J., Singh K. (2014). Microbial degradation of an organophosphate pesticide, malathion. Crit. Rev. Microbiol..

[2] Hamouda S.A., Marzouk M.A., Abbassy M.A., Abd-El-Haleem D.A., Shamseldin A. (2015). Isolation and identification of efficient Egyptian malathion-degrading bacterial isolates. J. Basic Microbiol..

[3] Pillans P.I., Stephenson B.A., Folb P.I. (1988). Cyclophosphamide effects on fetal mouse cephalic acetylcholinesterase. Arch. Toxicol..

[4] Kumar R., Nagpure N.S., Kushwaha B., Srivastava S.K., Lakra W.S. (2010). Investigation of the genotoxicity of malathion to freshwater teleost fish Channa punctatus (Bloch) using the micronucleus test and comet assay. Arch. Environ. Contam. Toxicol..

[5] Ahmed M., Rocha J.B., Mazzanti C.M., Morsch A.L., Cargnelutti D., Correa M., Loro V., Morsch V.M., Schetinger M.R. (2007). Malathion, carbofuran and paraquat inhibit Bungarus sindanus (krait) venom acetylcholinesterase and human serum butyrylcholinesterase In Vitro. Ecotoxicology.

[6] Gurushankara H.P., Krishnamurthy S.V., Vasudev V. (2007). Effect of malathion on survival, growth, and food consumption of Indian cricket frog (*Limnonectus limnocharis*) tadpoles. Arch. Environ. Contam. Toxicol..

[7] Fahmy G.H. (2012). Malathion Toxicity: Effect on Some Metabolic Activities in Oreochromis Niloticus, the Tilapia Fish. Int. J. Biosci. Biochem. Bioinform..

[8] Ullah S., Li Z., Hasan Z., Khan S.U., Fahad S. (2018). Malathion induced oxidative stress leads to histopathological and biochemical toxicity in the liver of rohu (*Labeo rohita*, Hamilton) at acute concentration. Ecotoxicol. Environ. Saf..

[9] Hoskin F.C.G., Walker J.E. (1997). Malathion as a Model for the Enzymatic Hydrolysis of the Neurotoxic Agent, VX. Bull. Environ. Contam. Toxicol..

[10] Asim N., Hassan M., Shafique F., Ali M., Nayab H., Shafi N., Khawaja S., Manzoor S. (2021). Characterizations of novel pesticide-degrading bacterial strains from industrial wastes found in the industrial cities of Pakistan and their biodegradation potential. PeerJ.

[11] Lauwers A.M., Heinen W., Gorris L.G., van der Drift C. (1990). Early stages in biofilm development in methanogenic fluidized-bed reactors. Appl. Microbiol. Biotechnol..

[12] Lewis D.L., Paris D.F., Baughman G.L. (1975). Transformation of malathion by a fungus, *Aspergillus oryzae*, isolated from a freshwater pond. Bull. Environ. Contam. Toxicol..

[13] Guha A., Kumari B., Bora T.C., Roy M.K. (1997). Possible involvement of plasmids in degradation of malathion and chlorpyriphos by *Micrococcus* sp.. Folia Microbiol..

[14] Mostafa I.Y., Fakhr I.M.I., Bahig M.R.E., El-Zawahry Y.A. (1972). Metabolism of Organophosphorus Insecticides XIII. Degradation of Malathion by *Rhizobium* spp.. Arch. Für Mikrobiol..

[15] Matsumura F., Boush G.M. (1966). Malathion degradation by *Trichoderma viride* and a *Pseudomonas* species. Science.

[16] Singh B., Kaur J., Singh K. (2012). Biodegradation of malathion by *Brevibacillus* sp. strain KB2 and Bacillus cereus strain PU. World J. Microbiol. Biotechnol..

[17] Singh B., Kaur J., Singh K. (2012). Transformation of malathion by *Lysinibacillus* sp. isolated from soil. Biotechnol. Lett..

[18] Tazdaït D., Abdi N., Lounici H., Grib H., Mameri N., Pauss A. (2013). Biodegradation of Malathion with Indigenous Acclimated Activated Sludge in Batch Mode and in Continuous-Flow Packed-Bed Reactor. Bioremediation J..

[19] Farag azmy A., Saafan A., Essam T., Amin M., Ahmed S. (2015). Biodegradation of Malathion by *Acinetobacter baumannii* Strain AFA Isolated from Domestic Sewage in Egypt. Int. J. Biol. Food Vet. Agric. Eng..

[20] Goda S.K., Elsayed I.E., Khodair T.A., El-Sayed W., Mohamed M.E. (2010). Screening for and isolation and identification of malathion-degrading bacteria: Cloning and sequencing a gene that potentially encodes the malathion-degrading enzyme, carboxylestrase in soil bacteria. Biodegradation.

[21] Khan S., Zaffar H., Irshad U., Ahmad R., Khan A., Shah M., Bilal M., Iqbal M., Naqvi T. (2016). Biodegradation of malathion by *Bacillus licheniformis* strain ML-1. Arch. Biol. Sci..

[22] Kim Y.H., Ahn J.Y., Moon S.H., Lee J. (2005). Biodegradation and detoxification of organophosphate insecticide, malathion by *Fusarium oxysporum* f. sp. pisi cutinase. Chemosphere.

[23] Sharma B., Dangi A.K., Shukla P. (2018). Contemporary enzyme based technologies for bioremediation: A review. J. Environ. Manag..

[24] Cho C.M., Mulchandani A., Chen W. (2004). Altering the substrate specificity of organophosphorus hydrolase for enhanced hydrolysis of chlorpyrifos. Appl. Environ. Microbiol..

[25] Bourquin A.W. (1977). Degradation of malathion by salt-marsh microorganisms. Appl. Environ. Microbiol..

[26] Janeczko A.K., Walters E.B., Schuldt S.J., Magnuson M.L., Willison S.A., Brown L.M., Ruiz O.N., Felker D.L., Racz L. (2014). Fate of malathion and a phosphonic acid in activated sludge with varying solids retention times. Water Res..

[27] Singh B., Kaur J., Singh K. (2013). Bioremediation of malathion in soil by mixed *Bacillus* culture. Adv. Biosci. Biotechnol..

[28] Moran M.A., Durham B.P. (2019). Sulfur metabolites in the pelagic ocean. Nat. Rev. Microbiol..

[29] Hucker G.J. (1921). A New Modification and Application of the Gram Stain. J. Bacteriol..

[30] Sohn J.I., Nam J.W. (2018). The present and future of de novo whole-genome assembly. Brief. Bioinform..

[31] Wayne L.G. (1988). International Committee on Systematic Bacteriology: Announcement of the report of the ad hoc Committee on Reconciliation of Approaches to Bacterial Systematics. Zent. Fur Bakteriol. Mikrobiol. Und Hygiene. Ser. A.

[32] Liu Y., Chen J., Jørgensen S.E., Fath B.D. (2008). Phosphorus Cycle. Encyclopedia of Ecology.

[33] Johan U.U.M., Rahman R.N.Z.R.A., Kamarudin N.H.A., Ali M.S.M. (2021). An integrated overview of bacterial carboxylesterase: Structure, function and biocatalytic applications. Colloids Surf. B Biointerfaces.

