# Supplementary material for: Isolation and Identification of Efficient Malathion-Degrading Bacteria from Deep-Sea Hydrothermal Sediment
Source: Microorganisms. 2022 Sep 6;10(9):1797. doi: 10.3390/microorganisms10091797 (PMC9502784; doi:10.3390/microorganisms10091797)
Supplement: Supplementary file 1 [file microorganisms-10-01797-s001.zip › microorganisms-1840877-supplementary.pdf]

1. Gram Staining of strain FG2 and strain CB1

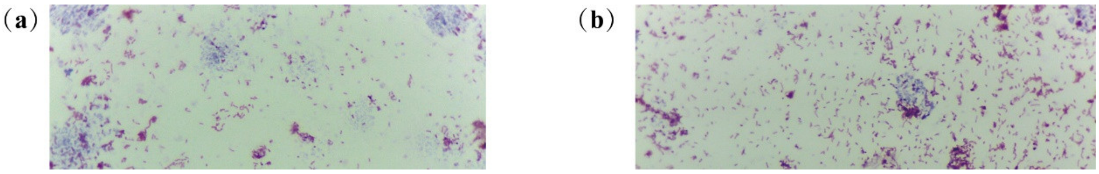

Figure S1. Gram staining of strain FG2 and strain CB1, (a) Gram-staining of strain FG2, Gram-negative rods. (b) Gram-staining of strain CB1, Gram-negative rods.

2. Scanning electron micrographs of strain FG2 and strain CB1 in different medium.

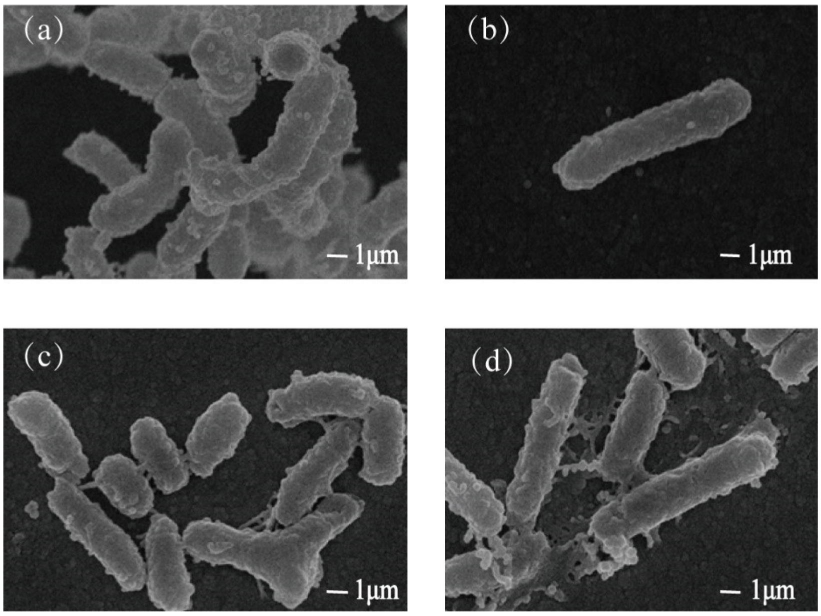

Figure S2. Scanning electron micrographs of strain CB1 in different medium for 10 h. (a) (b) Strain CB1 in 2216 E medium; (c) (d) Strain CB1 in M1 medium containing 500 mg/L malathion.

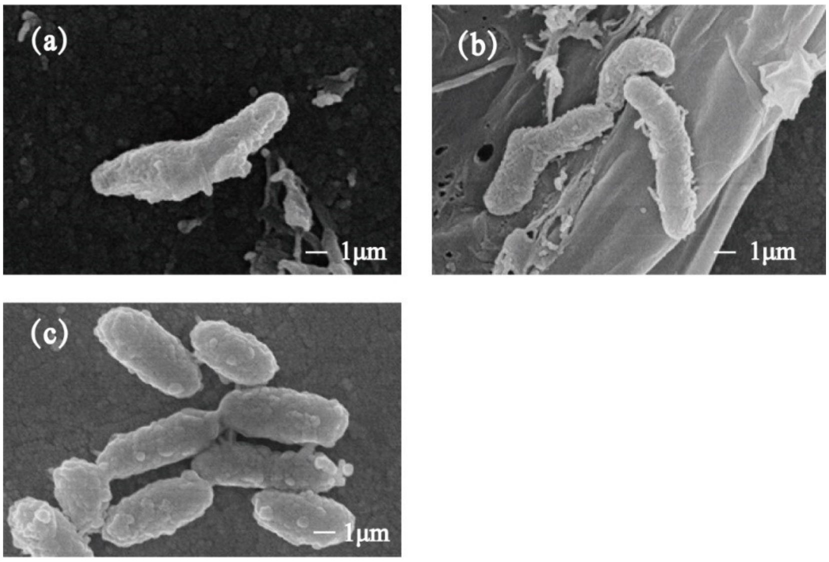

Figure S3. Scanning electron micrographs of strain FG2 in different medium for 10 h. (a) (b) Strain FG2 in 2216 E medium; (c) Strain FG2 in M1 medium containing 500 mg/L malathion.

3. Metabolites produced of malathion-degrading strain FG2 and strain CB1 by LC-MS/MS analysis

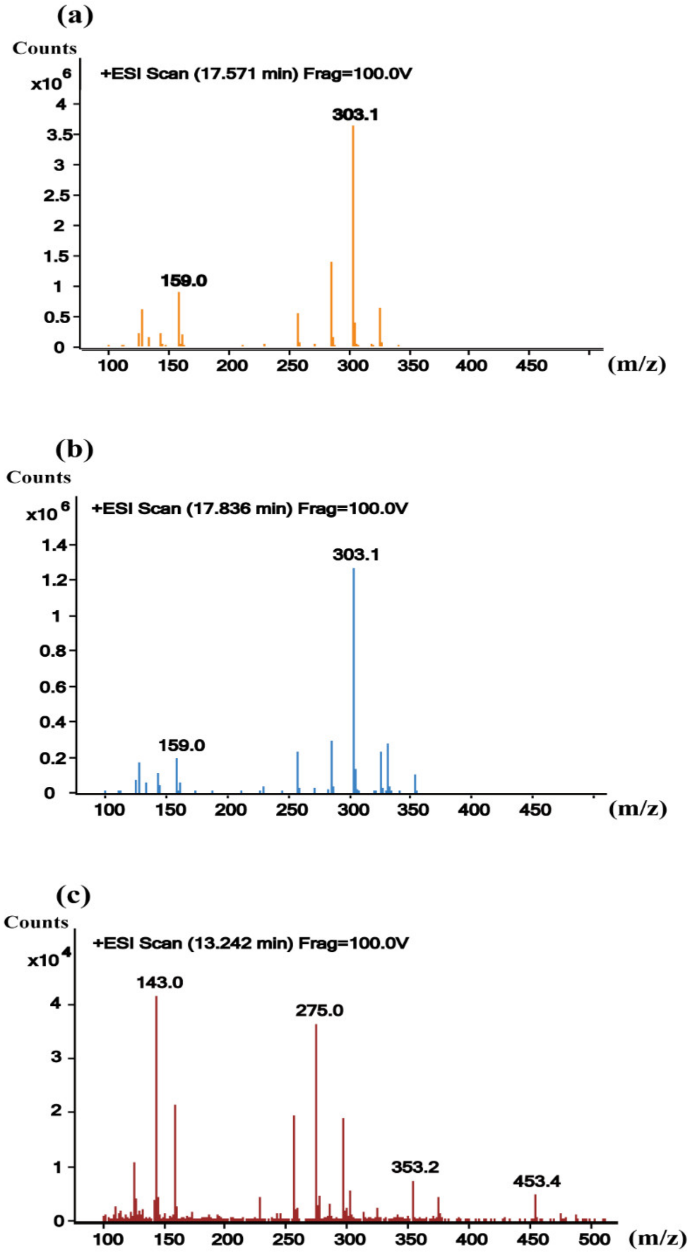

Figure S4. Mass spectrum chart of metabolites of malathion by strain FG2, (a) malathion monocarboxylic acid (α type); (b) malathion monocarboxylic acid (β type); (c) malathion dicarboxylic acid.

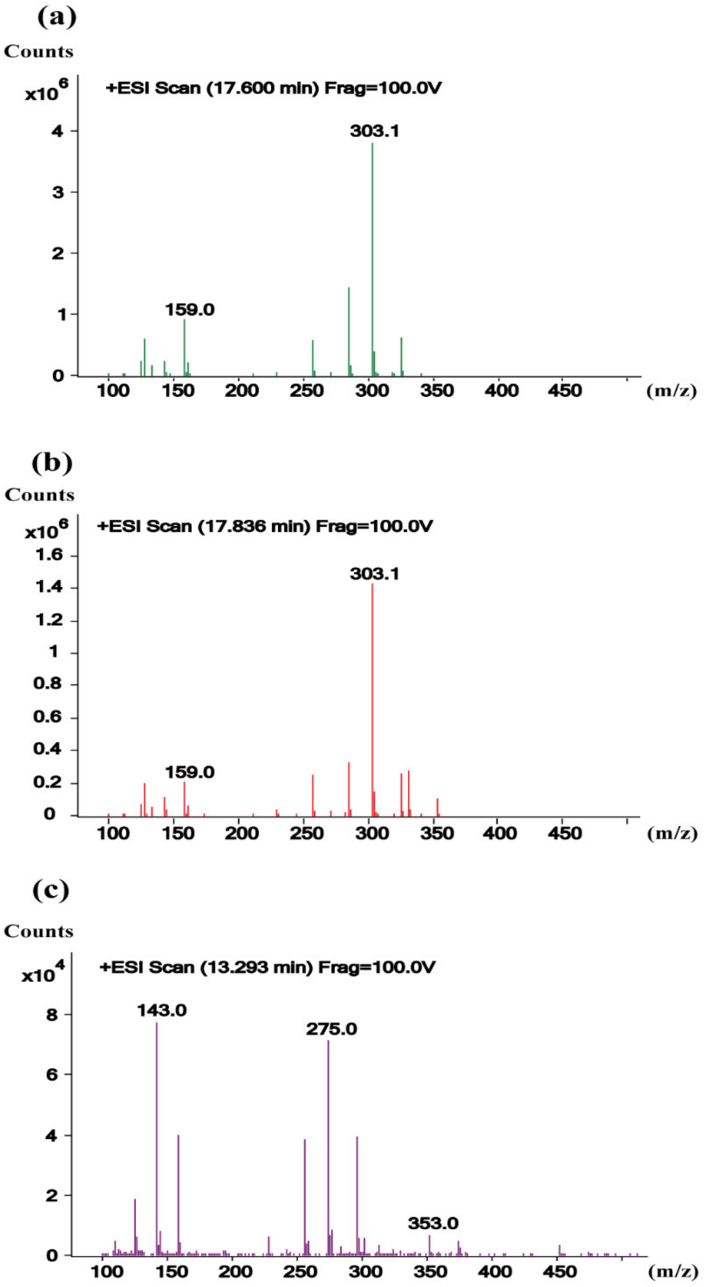

Figure S5. Mass spectrum chart of metabolites of malathion by strain CB1; (a) malathion monocarboxylic acid (α type); (b) malathion monocarboxylic acid (β type); (c) malathion dicarboxylic acid.

4. BioProject ID of strain FG2 and strain CB1:

FG2:

SUB11929399, PRJNA869452, SAMN30307835, JANQBU000000000.

CB1:

SUB11929382, PRJNA869451, SAMN30307798, JANTPU000000000
